# A Conceptual Model to Achieve Health Equity in APOL-1 Clinical Studies

**DOI:** 10.1089/heq.2024.0109

**Published:** 2024-09-26

**Authors:** Raven A. Hardy Richard, Rebecca Hayes, Robert Flemming, Cesia Portillo, Richardae Araojo, Christine Lee

**Affiliations:** ^1^ Office of Minority Health and Health Equity (OMHHE), Food and Drug Administration, Silver Spring, Maryland, USA.; ^2^ National Institutes of Health (NIH) National Human Genome Research Institute (NHGRI), Bethesda, Maryland, USA.; ^3^ North Carolina Community Health Center Association, Raleigh, North Carolina, USA.; ^4^ Department of the Air Force, Washington, District of Columbia, USA.; ^5^ American Society of Nephrology, Washington, District of Columbia, USA.

**Keywords:** APOL-1, clinical trials, health disparities, health equity, minority health

## Abstract

The socioecological model (SEM) conceptualizes health broadly and focuses on multiple factors that might affect health. This article takes a novel approach to leverage an SEM framework to identify challenges and sustainable opportunities to advance diversity for clinical study participation in apolipoprotein L1 (APOL-1)-mediated kidney disease. We describe four levels of an APOL-1 SEM—intrapersonal, interpersonal, community, and structures/systems—for improved diverse APOL-1 clinical study enrollment and engagement. This SEM can serve as a model for improving clinical study diversity and equity to help improve the generalizability of clinical study results in other disease conditions that disproportionally impact racial and ethnic minority populations.

## Introduction

Health equity is defined as “the state in which everyone has a fair and just opportunity to attain their highest level of health.”^1^ Equity is particularly important with respect to representation in clinical studies. Increasing diversity in clinical studies can strengthen generalizability, survival rate calculations, and estimates of treatment efficacy.^2^ It is important to continue to advance clinical study diversity for diseases that disproportionately impact racial and ethnic minority populations (e.g., kidney disease).

Individuals with recent African ancestry, including Hispanic/Latino populations, face a higher risk of chronic kidney disease (CKD) and end-stage kidney disease (ESKD) compared with those from other ancestries, even when factors such as socioeconomic status, lifestyle, and clinical factors are considered. The discovery of the apolipoprotein L1 gene (APOL-1) as a kidney disease risk gene revealed a genetic contribution to the racial and ethnic disparity observed in CKD.

Issues of health inequity do not exist in silos and are influenced by an individual’s connection to larger systems, such as family, caregiver, health care, and community. The socioecological model (SEM) proposes that an individual’s behavior is influenced by a dynamic network of intrapersonal characteristics, interpersonal relationships, community relationships, and structures/systems. SEM can be used to examine barriers associated with enrollment in clinical studies and lead to sustainable engagement in clinical studies.^3^ A novel approach to reduce inequities in APOL1-mediated kidney disease would be to adapt principles of the SEM to identify challenges and sustainable opportunities to advance diversity for APOL-1 clinical study participation to improve the generalizability of clinical study results.

We outline areas utilizing an SEM comprising four factors (intrapersonal, interpersonal, community, and systems/structures) that impact patients with APOL-1 and identify strategies to improve equity, recruitment, and retention in APOL-1 clinical studies (Table 1).

**Table 1.** Characteristics, Challenges, and Solutions within Levels of the Socioecological Model

SEM levels of support	Characteristics to consider	Challenges	Solutions
Intrapersonal Support			
	Culture and Language	The high prevalence of Latinos, speaking language, such as Spanish and Portuguese, which is impacted by APOL-1 necessitates developing strategies that account for individuals’ native language. The relationship between religion and health care impact the desire for racial and ethnic minorities to participate in clinical studies.	Access to bilingual clinical staff has shown to be a motivator for increased clinical study participation and enrollment. Interact with individuals at cultural centers to combat bias.
	Knowledge	APOL-1 and kidney disease is a life-altering disease which results in increased medical visits, treatments and therapies, and decreased quality of life resulting in an increase of fear, depression, anxiety, guilt, and a constant overwhelmed state. Disease status and comorbid diseases result in priorities that vary for individuals with high-risk APOL-1 alleles. Physical complications associated with dialysis or kidney transplantation, adverse reactions to therapies, and treatment complications.	Tailored education to address the unique needs at each level because individuals with developed diseases and comorbid disorders will have different desires and characteristics than those with risk alleles and no disease.
	Attitudes and Beliefs	Racial and ethnic minorities have a high desire to participate in clinical studies but are unsure of how to navigate trials. Patient and family involvement in the design and conduct of clinical studies is lacking. Individuals would like to know what benefits would be there.	Increase autonomy by engaging research participants in the planning, implementation, and dissemination of research. Increase patient and family involvement in the design and conduct of clinical studies. Increase patient autonomy to make informed decisions to let participate APOL-1 in clinical studies. Increasing participation of individuals from diverse backgrounds allows elevation of participants’ voices and experiences. Receive benefit from clinical trial.
	Behavior	A lack of access to information impacts the health behavior of patients.	Increase patient autonomy by understanding how their data will be used. Increase patient autonomy by understanding what clinical study results or data will mean to them.
Interpersonal Support			
	Family and Caregivers:	Family/Caregivers experience psychological distress, increased financial burden, and increased time commitment to care for individuals with APOL-1 and kidney disease. Family experience mixed emotions with knowledge that they could develop CKD and possess genetic risk alleles. Family members have expressed that they do not feel comfortable navigating clinical studies.	Engage family and caregivers in various phases of clinical study design and implementation. Consider the unique needs and impact of clinical study participation for family and caregivers.
	Support Groups:	Peer support and advice can influence participation in clinical trials. Shared experiences from others that have participated in trials can motivate or deter participation in trials.	Support groups, including social media groups, can be a resource to recruit and retain APOL-1 patients in clinical studies. Tele-health allows for remote support groups that allow engagement from those who could untraditionally not attend groups (i.e., those in rural areas).
	Community Health Workers (CHWs):	Resources and training about clinical trials and recruitment are limited for CHWs.	Support development of programs and culturally sensitive educational material with and for CHW to improve access and understanding of clinical study opportunities.
	Health Care Professionals (HCP):	Physicians are less likely to refer individuals of African descent to clinical studies. Physicians do not feel confident in their knowledge of APOL-1 to make recommendations. Physicians believe participation in trials would increase workload and negatively impact patient care.	Increase HCP awareness of APOL-1 education (CME, CEs, conferences). Clinical guidelines development for APOL-1 care HCP empowered to talk to their patients about clinical study participation. Increase training pathways for racial and ethnic minority HCPs for APOL-1 clinical studies. Tailored training should be developed on how to enroll and refer diverse participants to APOL-1 studies.
Community Support			
	Community Cornerstones	Being a part of an organized religion is associated with less desire to participate in clinical studies because of belief that community disapproves of participation. Communities do not desire to have relationships that do not offer a benefit to the community.	Leverage community cornerstones to improve population health outcomes. Support development of programs and culturally sensitive educational material with and for community cornerstones to improve access and understanding of clinical study opportunities.
	Safety Net Organizations:	There exists a lack of capacity to manage research activities, lack of ability to transform and share data, and lack of capacity to recruit research participants for safety net organizations.	Train providers and staff to promote clinical studies or conduct clinical research prioritize safety net organizations with enough support and resources to sustain efforts, including dedicated funding and staff.
System Support			
	Delivery Models	Issues related to funding and payment are often cited as barriers to individuals interested in participating in clinical research studies and payers only cover routine costs associated with participation in clinical studies.	Expanding VBP models to support provider and patient to participate in clinical studies. Leveraging APM incentives to support HCP to empower patients to participate in APOL-1 clinical studies.
	Stakeholder Engagement	Resources are often grant-related and unsustainable which amplify the distrust/mistrust within communities when resources run out and programs cease.	Strengthen engagement from government increases sustainability of clinical studies and increases the awareness of opportunities. Government funding for the recruitment and study of genetic contributors to kidney disease and the implementation of strategies such as those suggested in this article, potentially as criteria for funding support.

### Intrapersonal

Understanding the APOL-1 patient population is key to improving recruitment and retention in clinical studies. Acknowledging an individual’s culture, knowledge, attitudes, beliefs, and behavior would aid in sustainable engagement with APOL-1 clinical studies.

#### Culture and language

The high prevalence of Hispanic/Latino populations impacted by APOL-1^4^ necessitates developing strategies that account for individuals’ native speaking language, such as Spanish and Portuguese. Understanding cultural norms is also important, as factors such as religion and its relationship with health care impact the desire of racial and ethnic minorities to participate in clinical studies.^5,6^

#### Knowledge

One common theme among individuals with APOL-1 risk alleles is a desire for further education on APOL-1 and health.^7^ Disease status and stage vary for individuals with high-risk APOL-1 alleles resulting in different desires and characteristics; thus, education should be tailored to address the unique needs at each level. Knowledge of genetic risk increase the autonomy that patients feel when engaging with health care professionals (HCPs) and disclosure of genetic status increases desire for participation in research studies.^8^

#### Attitude and beliefs

Racial and ethnic minority populations have a high desire to participate in clinical studies.^9,10^ Engagement in clinical studies increases an individual’s sense of personal autonomy and their sustainable engagement in clinical studies.^11^ However, patient and family involvement in the design and conduct of clinical studies is still lacking, and strategies should be designed to increase patient engagement.^12^

#### Behavior

Individuals with and without kidney disease have consistently requested information on APOL-1 genetic knowledge to improve health behaviors.^13^ Providing APOL-1 genetic status during a clinical study has been shown to improve participant’s health outcomes and adoption of positive lifestyle changes. This subsequently led to an improvement of systolic blood pressure and increased compliance of urine testing for kidney function.^14^

### Interpersonal

Family, caregivers, and social networks are important entities for individuals with APOL-1, and patients are more likely to seek support for their medical conditions with individuals in their social network.^15^ Leveraging social networks can be beneficial in advancing diversity of research participants.

#### Family and caregivers

Family members have expressed that they do not feel comfortable navigating clinical studies, which motivates them to recommend against study participation.^12^ Special considerations should be given to the barriers to clinical study participation that caregivers experience, e.g., of as transportation to clinical studies, as caregivers will likely be providing most of the support for individuals with ESKD. Thus, it is important that family and caregivers are also engaged in the various phases of clinical study design and implementation.

#### Support groups

Support groups and peer systems improve health outcomes and quality of life for chronic conditions, such as kidney disease. Community members desire to be involved in the development of communication approaches and dissemination of APOL-1 materials.^16^ Support groups, including social media groups, can be a resource to recruit and retain APOL-1 patients in clinical studies.

#### Community health workers

Community health workers (CHWs), the frontline health workers who live in and are trusted by historically marginalized communities, are a unique part of a patient’s social network. One unique way to improve clinical study equity of the APOL-1 population, as well as other historically underrepresented populations, is to build on the growing success of CHWs to be part of the APOL-1 clinical study conversation and process.

#### Health care professionals

Patients with high-risk APOL-1 alleles and kidney disease interact with a variety of healthcare professionals (HCP) and those with good patient/HCP relationships will defer to HCP opinion about participation in clinical studies.^17^ Previous studies have noted that HCP are less likely to refer individuals of African descent to clinical studies.^18^ They do not feel confident in their knowledge of APOL-1 and desire more professional guidelines to make care recommendations about APOL-1.^19^ It is important to advance training and educational opportunities for HCP so that they can gain knowledge and the skills to advance APOL-1 clinical studies.

### Community

The idea of sustainable engagement with communities using existing community infrastructures is not a new one. Churches and barbershops are social networks that are traditionally seen as cornerstones in many communities.

#### Community cornerstones

Health promotion interventions based at churches and other community cornerstones, such as barbershops, are successful in reducing health disparities and improving health outcomes. Providing education on clinical studies and associated health conditions at churches increases desire to participate in studies, decreases anxiety about study participation, and provides an opportunity for feedback on making sessions more culturally competent.^20^

#### Safety net organizations

Federally qualified health centers (FHQC), rural health centers, public health departments, free clinics, and others serve the most diverse and vulnerable patient populations and have been embedded into the communities for decades. FQHC providers and staff are interested in providing their patients with access to clinical studies as well as participating in clinical research, but lack the funding and support needed to sustain their involvement.^21^

### Structures and systems

Many strategies have been developed to increase education and awareness of the importance of clinical study enrollment of representative populations, but these efforts have produced mixed results.^22^ These initiatives are oftentimes based on grant funding and created within separate infrastructures that rely on independent and irregular resources. To build trust and drive greater equity, we need to explore opportunities that can bridge education and clinical research with clinical care.^23^ Therefore, one idea is to work within existing systems to diversify and sustain engagement in clinical studies, such as insurance delivery models and collaboration with government stakeholders.

#### Delivery models

Issues related to funding and payment are often cited as barriers to individuals interested in participating in clinical research studies, especially those of lower socioeconomic status. Although clinical studies often support the direct costs associated with experimental treatments, indirect costs, such as childcare, are not supported. Payers can play a critical role in improving clinical care equity as most beneficiaries have some combination of limited income, time, and resources.

Value in health care is driven by the individual receiving care. In value-based payment (VBP), insurers create incentives, structured within alternative payment models (APM), for the health delivery system to work together to achieve the goals of the patient in the most efficient manner. Government and commercial insurance payers are increasing their utilization of different APM methodologies^24^ and creating opportunities to bridge the gap of systemic inequities with better access to care in populations most at need. VBP may offer an effective way to support equity in clinical study access through upfront payment to providers and support staff, such as CHWs. Providers can deliver information to patients about clinical studies as well as provide space to conduct research in more accessible locations to the communities who have historically been underrepresented.

#### Stakeholder engagement

Having engagement from the government increases sustainability of clinical studies and awareness of opportunities. Collaborations with government agencies and other stakeholders to develop clinical study resources can directly improve patient health. One successful initiative is the National Institutes of Health funded Kidney Precision Medicine Project which provides funding for the recruitment and study of genetic contributors to kidney disease.

## Conclusion

Implementing SEM in APOL-1 clinical studies provides a comprehensive and transformative approach to achieving health equity and outcomes by addressing barriers and opportunities at multiple levels—individual, interpersonal, community, and systemic. This approach aims to improve the generalizability of study findings, equip patients with knowledge and resources, and drive meaningful progress in reducing health disparities and advancing health equity.

By tailoring strategies to the unique cultural and social contexts of minority populations, enhancing the role of social networks, leveraging community resources, and integrating within existing health care structures, SEM can significantly improve the recruitment, retention, and overall engagement of racial and ethnic minorities in clinical trials resulting in direct and meaningful impact. For example, in a clinical study of patients with hypertension without kidney disease, the disclosure of high-risk APOL risk alleles status to patients led to the adoption of positive lifestyle changes. The return of results and increasing patient autonomy led to improved systolic blood pressure and increased compliance with urine testing for kidney function.^14^

We have proposed utilizing an SEM model to understand the multiple variables impacting patients with APOL-1 health journeys and offered solutions to increase and sustain participation in clinical studies. This strategy can be leveraged for other disorders to increase and retain diverse participation in clinical studies. However, it is essential to focus on the specific characteristics associated with each disorder to be genuinely beneficial.

## Author Disclose Statement

No competing financial interests exist.

## Abbreviations Used

APM: Alternative Payment Models
APOL-1: Apolipoprotein L1 Gene
CHWs: Community Health Workers
CKD: Chronic Kidney Disease
ESKD: End-Stage Kidney Disease
FQHC: Federally Qualified Health Centers
HCP: Health Care Professionals
SEM: Socioecological Model
